# 2019-nCoV: The Identify-Isolate-Inform (3I) Tool Applied to a Novel Emerging Coronavirus

**DOI:** 10.5811/westjem.2020.1.46760

**Published:** 2020-01-31

**Authors:** Kristi L. Koenig, Christian K. Beÿ, Eric C. McDonald

**Affiliations:** * County of San Diego, Health & Human Services Agency, Emergency Medical Services, San Diego, California; † University of California Irvine, Department of Emergency Medicine, Orange, California; ‡ University of California San Diego, La Jolla, California; § County of San Diego, Health & Human Services Agency, Public Health Services, San Diego, California

## Abstract

2019 Novel Coronavirus (2019-nCoV) is an emerging infectious disease closely related to MERS-CoV and SARS-CoV that was first reported in Wuhan City, Hubei Province, China in December 2019. As of January 2020, cases of 2019-nCoV are continuing to be reported in other Eastern Asian countries as well as in the United States, Europe, Australia, and numerous other countries. An unusually high volume of domestic and international travel corresponding to the beginning of the 2020 Chinese New Year complicated initial identification and containment of infected persons. Due to the rapidly rising number of cases and reported deaths, all countries should be considered at risk of imported 2019-nCoV. Therefore, it is essential for prehospital, clinic, and emergency department personnel to be able to rapidly assess 2019-nCoV risk and take immediate actions if indicated. The Identify-Isolate-Inform (3I) Tool, originally conceived for the initial detection and management of Ebola virus and later adjusted for other infectious agents, can be adapted for any emerging infectious disease. This paper reports a modification of the 3I Tool for use in the initial detection and management of patients under investigation for 2019-nCoV. After initial assessment for symptoms and epidemiological risk factors, including travel to affected areas and exposure to confirmed 2019-nCoV patients within 14 days, patients are classified in a risk-stratified system. Upon confirmation of a suspected 2019-nCoV case, affected persons must immediately be placed in airborne infection isolation and the appropriate public health agencies notified. This modified 3I Tool will assist emergency and primary care clinicians, as well as out-of-hospital providers, in effectively managing persons with suspected or confirmed 2019-nCoV.

## INTRODUCTION

2019 Novel Coronavirus (2019-nCoV) is a novel respiratory disease first reported in Wuhan, Hubei Province, China in December 2019.^1^ Chinese health officials were originally investigating a sudden increase in cases of pneumonia which were later determined to be linked to 2019-nCoV. While most cases originated within mainland China, the disease spread to neighboring countries including Taiwan, Thailand, South Korea, and Japan, and later to the United States, Europe, and Australia. A near real-time updated tracking website for cases and locations worldwide, along with reported deaths is available.^2^

Chinese health authorities have sequenced 2019-nCoV and freely shared its genetic profile online.^3^,^4^ Additionally, on January 28, 2020, an Australian laboratory reported growing the virus from a patient sample. As of January 30, 2020, there have been at least 9,776 persons infected and 213 verified deaths.^2^ These numbers are likely underestimates due to the limited information available regarding incubation time, transmissibility, and virus origin. The age distribution of these verified deaths is currently not available. One preliminary, small-scale study of 41 patients in Wuhan China, reported 6 deaths (15% mortality) with a median age of 49.0 years.^5^ Additionally, transmission of the virus has reportedly occurred in healthcare facilities in Wuhan City, raising concerns of spread to healthcare workers, as was seen during prior outbreaks of the novel coronaviruses, Middle Eastern Respiratory Syndrome (MERS) and Severe Acute Respiratory Syndrome (SARS). Due to the dynamic nature of the outbreak, exposure criteria may change depending on where new cases of 2019-nCoV are detected, the degree of transmissibility, and when additional information regarding the origin of the virus is discovered and reported.

On January 15, 2020, the Centers for Disease Control and Prevention (CDC) confirmed the first known imported case of 2019-nCoV in the US state of Washington. The patient had recently returned from Wuhan City, where he likely contracted the disease. Chicago health authorities reported a second US case on January 24, 2020. This was quickly followed by additional imported cases reported in Orange and Los Angeles Counties, California on January 26, 2020. Additional suspected cases continue to be evaluated. On January 30, 2020, the CDC reported the first local transmission in the US between members in a household. On the same day, the World Health Organization declared 2019-nCoV to be a Public Health Emergency of International Concern (PHEIC).^6^ On January 31, 2020, the US Department of Health and Human Services declared coronavirus a public health emergency.^7^

Healthy individuals and those with mild illness may be asymptomatic, while others may have more pronounced symptoms of fever or lower respiratory illness. Upon identification of a suspected patient, that individual should immediately be isolated with airborne precautions. Further work-up and laboratory confirmation can then proceed. Emergency physicians (EPs), emergency medical services (EMS) personnel, and other healthcare workers who encounter patients with suspected 2019-nCoV infection must inform the appropriate authorities, including but not limited to hospital infection control and local or state public health agencies.

Healthcare workers must follow on-going developments related to the outbreak, especially new information concerning detection and management.^8^,^9^ The 3I Tool outlined in this paper is consistent with current US CDC guidelines and can be applied in a variety of settings such as those in emergency departments, urgent-care clinics, physicians’ offices, and prehospital settings. This paper will first briefly review 2019-nCoV and then present the novel 2019-nCoV 3I Tool as modified from its initial conception for Ebola virus disease 10,11 and later adapted for measles,^12^ MERS,^13^ mumps,^14^ Zika virus disease,^15^ hepatitis A,^16^ pertussis,^17^ and scabies.^18^

## CLINICAL PRESENTATION

### Signs and Symptoms

Coronavirus 2019-nCoV infection commonly presents with signs and symptoms of pneumonia or as a nonspecific lower respiratory illness, with coughing or difficulty breathing accompanied by fever.^5^,^19^,^20^ Fever and cough constitute the most common presentations. However, patients may have other respiratory symptoms, sore throat, nasal congestion, malaise, myalgia, and headache. Bilateral infiltrates may be seen on chest X-ray. Severe cases may present with sepsis and even shock. Conversely, some patients may present as only mildly ill or asymptomatic altogether.^21^ To date, patients with underlying medical conditions and the elderly are more likely to become severely ill, require hospitalization, and ultimately die.^22^ Early predictions for incubation time are between 2 and 14 days, based on data from similar coronaviruses. The 14-day criterion for epidemiological risk assumes the longest estimated incubation time.^23^ In addition, the World Health Organization (WHO) has created its own interim case definition.^24^

**Population Health Research Capsule**

What do we already know about this issue?

2019 Novel Coronavirus (2019-nCoV) is a rapidly spreading infectious disease closely related to severe acute respiratory syndrome (SARS)-CoV and Middle East respiratory syndrome (MERS)-CoV, first detected in late 2019 in Wuhan, China.

What was the research question?

Investigators adapted the “Identify, Isolate, Inform” (3I) Tool for use in suspected cases of 2019-nCoV.

What was the major finding of the study?

A novel 2019-nCoV 3I Tool is designed for frontline clinicians in the management of suspected patients.

How does this improve population health?

This 2019-nCoV 3I adaptation will aid healthcare providers most likely to encounter the disease in the containment and effective treatment of patients.

### Disease Characteristics

By definition, the main features of a novel virus, for example, how it is transmitted, will not be immediately known. However, as with the development of any 3I Tool, it is essential to understand specific characteristics of the disease. In the case of a novel virus such as 2019-CoV, this is challenging since information is rapidly evolving and the science is not yet fully understood. It is possible that the virus will undergo mutations over time that could substantially change its features. Nevertheless, an appreciation of the key concepts that drive evidence-based management is beneficial (Table 1). Management guidance will likely change over time.

With the initial discovery of a new potential public health threat, it will likely be unclear how patients become sick. For example, rather than a contagion, there could be a contaminant or a toxin responsible for signs and symptoms. In this case, the possibility of an environmental toxin in the Wuhan Market was a consideration early on when limited to no human-to-human transmission was reported. The mode of transmission has implications for the types of personal protective equipment (PPE) needed to protect healthcare providers in the prehospital, clinic, and hospital settings.^25^ In addition, patients may need decontamination after exposure to certain toxins.^26^

Another important consideration for application of the 3I Tool is whether the disease is contagious prior to symptom onset (like measles) or only after symptoms develop (like Ebola). A January 30, 2020 letter to the *New England Journal of Medicine* describes a purported confirmed instance of transmission from an asymptomatic individual. Researchers state that, before symptom onset, the primary case infected two individuals, one of which infected two additional colleagues.^27^ Subsequent investigation suggested that the source patient did have mild symptoms and had taken an antipyretic, calling this reported asymptomatic transmission into question.

While quarantine may not be feasible and can have unintended consequences,^28^,^29^,^30^ it is a public health tool that can be considered in cases when disease is transmissible before symptom onset.^30^ Conversely, if a disease is known not to be transmissible prior to symptom onset, asymptomatic exposed patients must be monitored, but do not require quarantine or isolation unless they develop symptoms.

Initially, it may be unclear whether an infectious agent occurred naturally or was deliberately or accidentally released. In this case, a BSL-4 laboratory studying coronaviruses was located approximately 32 kilometers away from the market where initial exposures were felt to occur.^31^ Recall that in 2001, the anthrax letter attacks were initially thought to be naturally occurring. Once determined to be bioterrorism, management of the event was similar to that for a chemical exposure with a sudden impact, defined scene, and need for a rapid response and decontamination on site. This differed from the WHO’s modeling predicting an aerosolized release that would result in an incubation period with 100,000 or more persons exposed rather than the 22 people who contracted anthrax in 2001.^32^ By understanding the key features of a novel disease, healthcare workers can take evidence-based measures to protect themselves, optimize individual patient management, and prevent further disease spread.

### Transmission

It is currently unclear how 2019-nCoV is spread, but it is suspected to be transmitted through contact with infected respiratory secretions, like other known coronaviruses. There are instances of sustained human-to-human transmission across generations of cases, especially near the epicenter in Wuhan City.^21^ Current evidence suggests that close contact with an infected person is a major factor in disease transmission. CDC defines “close contact”^33^ as being in or within two meters of an area with a confirmed patient or being directly exposed to infectious secretions without appropriate PPE. Healthcare facilities in China have reported spread from person to person. In addition, some mildly ill or potentially even asymptomatic patients may have a higher chance of spreading the disease to others as they may be less likely to seek medical care.^34^ The possibility that patients may be infectious prior to symptom onset further compounds the difficulty of containing the virus and effectively preventing transmission.

The current majority of 2019-nCoV cases have been within China and its bordering countries.^2^ Persons with recent travel (within 14 days) to Wuhan City or another region with widespread disease, or exposure to a patient under investigation, are considered to have an epidemiologic risk factor and should be assessed for signs and symptoms of a viral illness such as fever and respiratory symptoms. Coronavirus is a zoonotic virus that is transmitted to humans via contact with infected animals. Preliminary reports suggest the disease may have originated in a seafood and live animal market in Wuhan City, but it is still unknown how or whether such transmission occurred.

### Work-Up

Clinicians working with local public health departments must arrange to have specimens from patients under investigation (PUIs) sent to the CDC laboratory. At this time, the CDC has the only laboratory that can definitively test for 2019-nCoV, though laboratory testing capacity is being rapidly expanded. Polymerase chain reaction (PCR) assays conducted on samples from a patient’s upper and lower respiratory tracts will be used to confirm potential cases. In addition, serum antibody titers can be analyzed for confirmation of infection or evidence of immunity. Up-to-date information about the needed specimens and handling requirements to test for 2019-nCoV are available on the CDC website.^35^

### Differential Diagnosis

Like other related coronaviruses, patients with 2019-nCoV frequently present with non-specific symptoms resembling that of influenza. Physicians may consider differential diagnoses related to a wide variety of respiratory infections. In order to relate these symptoms to 2019-nCoV, it is imperative that the identification of a potential exposure event (epidemiologic risk factor) within 14 days of symptom onset is made so that a more focused work-up for 2019-nCoV can be completed. Although the likelihood of co-infection of 2019-nCoV and another respiratory virus is thought to be low, a positive finding of another respiratory pathogen does not exclude the diagnosis of 2019-nCoV. Many commercially available respiratory panels include “coronavirus” in the results, but neither a positive nor a negative finding on these panels should be used to include or exclude a diagnosis of 2019-nCoV.

### Treatment

Supportive care with appropriate infection control is the mainstay of current CDC treatment guidelines for 2019-nCoV. There are not yet any approved antiviral treatments for 2019-nCoV. Emergency Use Authorizations (EUA) for compassionate use cases may be forthcoming from the US federal government for normally unapproved treatments. Supportive treatment predominantly includes respiratory support, hydration, and antipyretics. General treatment for severe cases should focus on the preservation of vital organ function. In the future, antiviral medications may be available. If a secondary bacterial infection such as pneumonia develops, targeted antibiotics are indicated.

### Prevention

Prevention of 2019-nCoV transmission, like any other infectious agent, involves minimizing risk of exposure. Vaccines are under accelerated development and may be useful in the future for post-exposure prophylaxis. Healthcare personnel are at increased risk and should practice standard, droplet, and airborne precautions when encountering an infected person, a PUI, or any symptomatic close contacts. Healthcare workers handling specimens should also adhere to CDC guidelines and should not attempt to perform any virus isolation or characterization.

Fever screening has been implemented at numerous airports, including major international hubs within Asia and the US. The efficacy of this intervention is not well documented, however, as some infected persons may be afebrile and disease transmission might occur prior to symptom onset.^27^ In addition, people can artificially lower their temperature readings, e.g., by applying ice to their foreheads.

### Patient Disposition

As outlined above, admission criteria for 2019-nCoV are similar to that of other patients. If patients do not meet medical criteria for hospitalization, they may be discharged home with isolation precautions and continued observation. EPs must notify local public health authorities so appropriate monitoring and community protective measures can be instituted.

### Identify-Isolate-Inform

**Figure 1:**
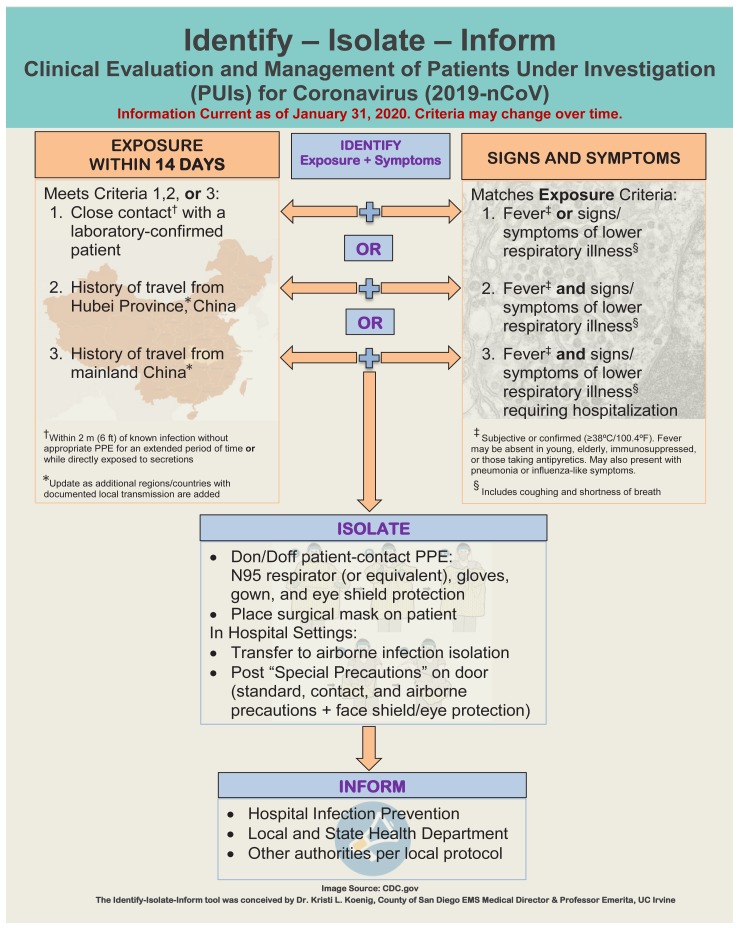
Koenig’s Identify-Isolate-Inform Tool adapted for 2019-nCoV.

The Identify-Isolate-Inform (3I) Tool was initially developed for Ebola virus disease 10,11 and later adapted for measles,^12^ MERS,^13^ mumps,^14^ Zika virus disease,^15^ hepatitis A,^16^ pertussis,^17^ and scabies.^18^ This novel tool for suspected 2019-nCoV patients (Figure 1) provides frontline clinicians with a simple algorithm to manage an emerging disease. Identification of exposed patients with an epidemiologic risk factor within 14 days of symptom onset is a crucial first step. An automatic prompt in the electronic health record can be useful in assisting clinicians with early identification of patients at risk. Case definitions promulgated by the WHO^24^ and CDC^33^ provide useful comprehensive definitions that have been incorporated into the 3I Tool. The 2019-nCoV Tool provides an accurate, summarized algorithm to immediately, and effectively manage suspected patients until additional resources can be consulted.

Patients who do not have an exposure risk or any symptoms may be triaged normally. However, before making patient contact, providers must first apply the Vital Sign Zero concept.^36^ Vital Sign Zero is a preliminary, non-contact assessment (i.e., performed prior to touching a patient to take traditional vital signs) to first determine whether specific PPE is indicated before the examination commences. By taking the additional time to complete this assessment, risk of exposure and further transmission can be minimized.

Following identification of patients with clinical features and an established epidemiologic risk, isolation should occur immediately. Patients should don a surgical mask before being placed in an airborne infection room, if available. Any healthcare staff entering the room should don a NIOSH-certified N95 respirator (or equivalent), eye protection, gloves, and a gown, as per CDC recommendations. Further patient contact and sample collection may then proceed. A list of all individuals (staff or other patients) who were in close contact with the individual while in the treatment facility should be started and maintained to assist with the possibility of contact tracing.

Following isolation, physicians should immediately inform the appropriate authorities. Patients who do not meet medical criteria for admission can be isolated at home during the evaluation phase.^37^ Health department officials can help prevent transmission in isolated patients by providing in-home monitoring and implementing appropriate exposure-control measures.

### Prehospital EMS Management

Providers in the prehospital setting who have a high likelihood of encountering 2019-nCoV patients, such as those near international ports of entry, should adhere to established exposure control guidelines.^38^ Along with appropriate PPE, providers should also carry thermometers to quantify any fever. In the US, providers should contact the appropriate CDC quarantine station upon isolation of infected or suspected patients, especially those from Wuhan, China or other regions with widespread disease, who report symptoms in the last 14 days. As for other infectious diseases, assessing travel history is essential. Dispatch protocols have been instituted to facilitate identification of callers to 911 or the country-equivalent emergency number prior to prehospital personnel arrival.^39^ In addition, CDC has promulgated EMS guidelines for prehospital PPE, transportation of PUIs, vehicle decontamination, and 911 Public Safety Answering Points (PSAPs) for 2019-nCoV.^40^

## CONCLUSION

2019-nCoV is an emerging infectious disease with rapidly evolving features, the full scope of which will be defined over time. Prior outbreaks of coronaviruses can help inform needed actions in the short term to assist with both treatment of individual patients and prevention of global disease spread. This adaptation of the Identify-Isolate-Inform Tool serves as a resource for healthcare workers who need to make clear, rapid assessments when confronted with potential patients. The concise nature of the 2019-nCoV 3I Tool allows for the rapid and effective management of a novel disease by healthcare providers.

## Figures and Tables

**Table 1:** Key disease features and implications.

Scenario	Special Considerations
Natural vs. human-generated (e.g., terrorism, industrial incident)	Law enforcement in addition to public health investigation; crime scene investigation
Contagion vs. contaminant/toxin vs. neither	Mode of transmission; PPE type
Transmissibility from person to person (i.e., R_0_)	PPE requirements; need for declaration of PHEIC, need for surge capacity
Potential for mutations	Need for monitoring and updates on public health management guidance
Sensitivity and specificity of testing	Strategies for testing method and location (point-of-care, regional, national)
Contagious prior to symptom onset	Amenable to quarantine; types of public health interventions needed to prevent spread

*PHEIC*, Public Health Emergency of International Concern; *PPE*, personal protective equipment; *R_0_*, Basic Reproduction Number: a mathematical prediction of disease contagiousness.
